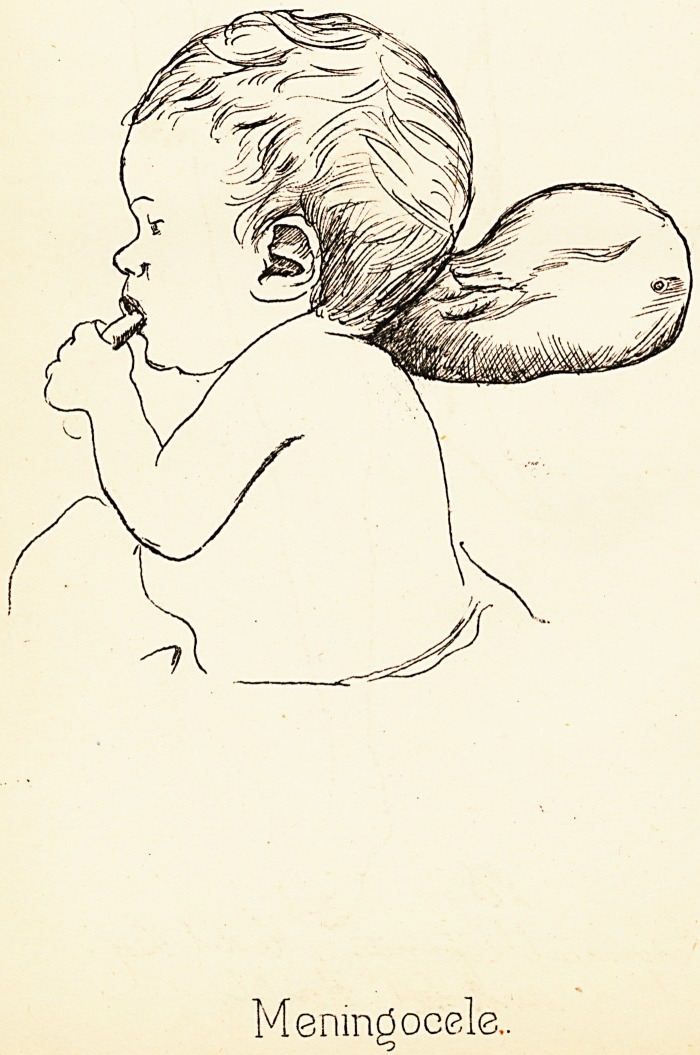# Case of Very Large Meningocele

**Published:** 1883-07

**Authors:** 


### CASE OF VERY LARGE MENINGOCELE.

This case is recorded on account of the large size to which the tumour grew without seriously affecting the health of the child. It lived till it was nearly a year and a half old. The accompanying drawing is traced from a photograph (Pl. X.). The growth protruded from an opening in the occipital bone, and was covered partly by the skin of the neck and partly by the scalp.

## Figures and Tables

**Figure f1:**